# The Role of Natural Flavonoids as Telomerase Inhibitors in Suppressing Cancer Growth

**DOI:** 10.3390/ph16040605

**Published:** 2023-04-17

**Authors:** Neel Parekh, Ashish Garg, Renuka Choudhary, Madhu Gupta, Ginpreet Kaur, Seema Ramniwas, Moyad Shahwan, Hardeep Singh Tuli, Gautam Sethi

**Affiliations:** 1 Shobhaben Pratapbhai Patel School of Pharmacy & Technology Management, SVKM’S NMIMS, Vile Parle (W), Mumbai 400056, India; 2 Department of P.G. Studies and Research in Chemistry and Pharmacy, Rani Durgavati University Jabalpur, Jabalpur 482001, India; 3 Department of Biotechnology, Maharishi Markandeshwar, Deemed to be University, Ambala 133207, India; 4 Department of Pharmaceutics, School of Pharmaceutical Sciences, Delhi Pharmaceutical Sciences and Research University, Pushp Vihar, New Delhi 110017, India; 5 University Centre for Research and Development, University Institute of Pharmaceutical Sciences, Chandigarh University, Gharuan, Mohali 140413, India; 6 Department of Clinical Sciences, College of Pharmacy and Health Sciences, Ajman University, Ajman 346, United Arab Emirates; 7 Centre of Medical and Bio-Allied Health Sciences Research, Ajman University, Ajman 346, United Arab Emirates; 8 Department of Pharmacology, Yong Loo Lin School of Medicine, National University of Singapore, Singapore 117600, Singapore

**Keywords:** telomerase, anti-cancer, natural flavonoids, luteolin, hTERT

## Abstract

Cancer is a complex and multifaceted group of diseases characterized by the uncontrolled growth and spread of abnormal cells. While cancer can be challenging and life-altering, advances in research and development have led to the identification of new promising anti-cancer targets. Telomerase is one such target that is overexpressed in almost all cancer cells and plays a critical role in maintaining telomere length, which is essential for cell proliferation and survival. Inhibiting telomerase activity can lead to telomere shortening and eventual cell death, thus presenting itself as a potential target for cancer therapy. Naturally occurring flavonoids are a class of compounds that have already been shown to possess different biological properties, including the anti-cancer property. They are present in various everyday food sources and richly present in fruits, nuts, soybeans, vegetables, tea, wine, and berries, to name a few. Thus, these flavonoids could inhibit or deactivate telomerase expression in cancer cells by different mechanisms, which include inhibiting the expression of hTERT, mRNA, protein, and nuclear translocation, inhibiting the binding of transcription factors to hTERT promoters, and even telomere shortening. Numerous cell line studies and in vivo experiments have supported this hypothesis, and this development could serve as a vital and innovative therapeutic option for cancer. In this light, we aim to elucidate the role of telomerase as a potential anti-cancer target. Subsequently, we have illustrated that how commonly found natural flavonoids demonstrate their anti-cancer activity via telomerase inactivation in different cancer types, thus proving the potential of these naturally occurring flavonoids as useful therapeutic agents.

## 1. Introduction

Irreversible impairment of cellular homeostasis triggers the heterogeneous disease called cancer. Uncontrolled cell growth and differentiation, in addition to the loss of apoptotic functions, leads to cancer progression [1]. In 1989, telomerase was investigated in the transformed cervical carcinoma cell line [2]. Telomerase is a unique reverse transcription enzyme that connects tandem repeats at the 3′ ends of chromosomes, which produce the telomeres and reimburse the loss of those telomeric sequences. By using an RNA template, this enzyme adds nucleotide repeats to telomeres, thereby compensating for the loss of DNA replication and providing karyotype stability [3]. In about 85–90% of cases, telomerase is predominantly expressed in tumor cell lines and human tumors and is responsible for maintaining telomere length [4,5,6]. The main role of this enzyme is telomere capping and responding to DNA damage [7,8]. Telomerase plays a crucial role in cancer cell development and is generally observed in most cancer cells. Therefore, the inhibition and activation of telomerase are essential for the cancer regulation (activation or suppression) mechanism [9]. During malignancy, telomerase ensures indefinite cell proliferation and can act as a preferred target for drug development in cancer therapy [10,11,12].

**Figure 1:**
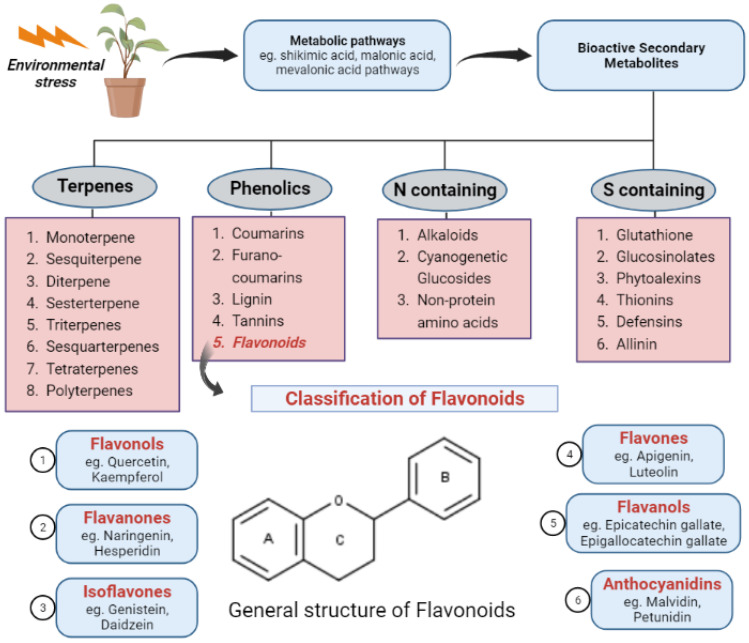
Classification of plant secondary metabolites and flavonoids.

A rising trend in the incidence of cancer has been consistently observed, and it is a chief contributor to mortality at the global level. The number of new cancer cases reported annually is speculated to increase to 29.5 million by 2040. By the same time, cancer deaths are predicted to be 16.4 million annually. Ranked from highest to lowest, the most common cancer types include those of breast, lung, bronchus, prostate, colon and rectum, skin, bladder, kidney, endometrium, thyroid, and liver [13]. Breast and lung malignancies are the two most commonly occurring cancers worldwide, with a 12.5% and 12.2% contribution to new cancer cases in 2020, respectively. These are followed by colorectal cancer, contributing 10.7% of new cases in 2020 [14]. In the present era, for the treatment of cancer, synthetic compounds are commercially used as chemotherapeutic agents. However, most have numerous side effects. Therefore, natural products are explored for their beneficial effects as anti-cancer therapy [15,16,17]. Natural products are normally taken in human diets and as functional foods that inhibit telomerase activity in cancer patients [18]. Plants synthesize a wide array of bioactive secondary metabolites that are produced due to external stimuli, such as environmental stress, and they offer a physiological function by providing structural support to the plant itself, attracting pollinators, etc. Various metabolic pathways, such as the shikimic acid, malonic acid, and mevalonic acid pathways, are involved in the synthesis of secondary metabolites. More than 200,000 metabolites have been identified and are majorly classified as nitrogen-containing, sulfur-containing, terpenes, and phenolics [19] (refer to Figure 1). These metabolites have numerous pharmacological, nutraceutical, and cosmetic applications. Many of these secondary metabolites possess anti-cancer activity and have led to researchers screening these phytochemicals to develop novel agents for effective anti-cancer benefits. For instance, paclitaxel, vincristine, homoharringtonine, curcumin, resveratrol, and betulinic acid are some well-established anti-cancer agents [20].

Similarly, flavonoids are polyphenolic compounds synthesized as bioactive secondary metabolites [21]. Their main sources are fruits and vegetables [22,23,24]. Flavonoids are classified as flavonols, flavanones, flavanols, flavones, anthocyanidins, as well as isoflavonoids and are further divided into subgroups. Numerous studies at in vitro and in vivo levels have confirmed the activity of flavonoids against various cancer cell lines [25,26,27]. The numerous anti-cancer effects of flavonoids have been reported by different studies, such as reactive oxygen species (ROS)-scavenging enzyme activities modulation, cell cycle arrest participation, apoptosis, and cancer cell proliferation suppression [28,29]. Chemical structure and hydroxyl group substitution of flavonoids are the major factors on which the radical scavenging activity of the class of compounds is dependent. SAR studies suggest the significant role of phenolic OH functional groups (their number and location), which govern the anti-radical potential. The catechol structure in ring B, electron-donating properties, and offering as a radical target are the structural requirements for effective radical scavenging activity. Even the 3-OH group in the C-ring enhances the antioxidant activity of flavonoids [30]. Plant secondary metabolites play a crucial role in reducing telomerase activity and inducing apoptosis. Numerous studies have shown that secondary metabolites have the potential to inhibit telomerase activity and induce apoptosis [31,32].

Thus, the main aim of this review is to present comprehensive details and put forward the use of flavonoids as phytomedicine that inhibits telomerase activity, leading to cancer prevention, and can be used as a target in the future for cancer therapy. As no literature has emphasized both flavonoids and telomerase inhibition, this review will serve as a cue to initiate detailed research for cancer therapy and give the readers a strong foundation on the discussed topic. The review predominantly covers relevant data and information from 2014–2023.

## 2. Telomerase as a Potential Target for Cancer Therapy

Carcinoma is often a hereditary disorder associated with aging that only becomes visible when normal cells get the capacity to replicate immortally and amass genomic instability. When cells divide repeatedly, telomere attrition causes chromosomal instability and makes a substantial contribution to the genomic rearrangements that may lead to cancer. The survival of cancer cells depends on telomeres, which are repeating “(TTAGGG) DNA–protein complexes” near the ends of chromosomes. In the great majority of cancers, an enzyme known as telomerase keeps them intact. “Telomere length (TL)” maintenance and telomerase expression are regulated by epigenetic, posttranscriptional, and transcriptional phenomena, and a thorough understanding of these mechanisms may lead to the discovery of new biomarkers and therapeutic targets for the early diagnosis of disease, the assessment of the prognosis of the condition, and the development of new treatments [8,33].

Since telomerase is present in most carcinoma cells, in addition to cancer stem or stem-like cells, it has been a primary target for the production of potent anti-cancer treatments. Additionally, telomerase expression is lower in normal human cells, particularly stem cells, and they typically keep their telomeres longer than cancer cells do [34,35]. These characteristics provide a benefit that assures a low risk of potential telomere shortening in healthy cells. Anti-telomerase treatments’ primary goal is to selectively cause apoptosis and cell death in cancer cells while minimizing the consequences for healthy cells [36,37].

This objective has been attained via a variety of strategies, including the creation of vaccinations, antisense oligonucleotides, and small-molecule antagonists that target the human telomerase RNA component (hTR) or human telomerase reverse transcriptase (hTERT). Even though Bryan and colleagues [38] observed an innovative telomerase inhibitor, BIBR1532, that adheres to the thumb domain of TERT and disrupts TERT–RNA interaction (telomerase ribonucleoprotein assembly), resulting in the suppression of enzymatic activity, oligonucleotide imetelstat (GRN163L) appears to be the most promising telomerase inhibitor. However, clinical studies for this substance have not yet advanced.

Furthermore, the advancement of G-quadruplex stabilizers and tankyrase inhibitors (which play a significant role in WNT/β-catenin signaling, mitotic spindle formation, and telomere function) and HSP90 inhibitors (which are involved in signaling pathways, signal transduction, and protein breakdown), designed to target telomere and telomerase construction, in addition to T-oligo (DNA oligonucleotide homologous to the telomere 3′ overhang region, which causes cytotoxic effects by inducing DDR), have also been explored to selectively kill cancer cells [39].

In contrast, immune-based treatments that make use of dendritic cells (GRVAC1), hTERT peptides (GV1001), or cryptic peptides (Vx-001) are now undergoing testing in experimental studies. Imetelstat is the only anti-telomerase chemical that has been subjected to comprehensive testing in investigational studies. Many anti-telomerase therapies, including vaccines, are now undergoing various stages of clinical testing; nevertheless, imetelstat is the only anti-telomerase agent that has been tested. Recently completed clinical investigations targeting telomerase for cancer treatment are mentioned in Table 1.

Telomerase has been regarded as an appealing target for the treatment for cancer since it was discovered, over two decades ago, that the stimulation of such enzymes in cancerous cells promotes immortalization through telomere expansion [45]. Natural products can effectively modulate the different hallmarks of cancer cells through diverse molecular mechanisms [46,47]. Numerous plants and their parts, such as leaves and fruits and vegetables, may contain phytoconstituent molecules, called flavonoids, that have significant uses in medical biochemistry. Flavonoids provide a variety of health advantages, such as anti-inflammatory, antioxidant, anti-cancer, and anti-viral effects. Additionally, they possess neuroprotective as well as cardioprotective properties [48].

Some flavonoids have a stunning array of biological uses and health-improving qualities. They have drawn significant interest since their widespread use is supported by several epidemiological investigations [49]. In addition, the activation of telomerase in precursor and stem cells, such as those that give rise to hematopoietic lineages, is a significant source of adverse reactions brought on by treatments that target telomerase [50]. 

**Figure 2:**
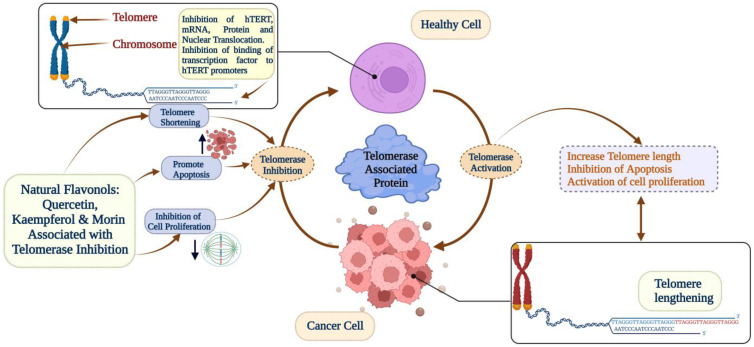
Telomerase-associated anti-cancer activity of natural flavonoids.

Furthermore, flavonoids have the potential to be used as telomerase inhibitors and powerful candidates for suppressing the function of telomerase in order to stop the growth of tumor cells and start the apoptotic process. There is a possibility that flavonoids such as epigallocatechin gallate, morin, kaempferol, quercetin, luteolin, and apigenin might be agents that suppress the action of telomerase. The telomerase-associated anti-cancer activity of natural flavonoids is illustrated in Figure 2.

Despite the fact that telomerase offers a number of advantageous characteristics for the progression of tumors, effective therapeutic treatments have been severely constrained by problems with pre-clinical animal models, a lack of a high-resolution structure of human telomerase, and adaptive pharmaceutical tolerance. Because they are dependent on the progressive shortening of telomeres that occurs with every cellular division, therapeutic interventions that are based on inhibiting the function of telomerase reverse transcriptase require a prolonged period of intervention before the anti-tumor effects are imposed. This is because of the nature of their mechanism. Because of this, it is possible that they cannot be used as a first-generation treatment, and it also means that there is a greater chance of resistant copies evolving and spreading.

## 3. Flavanols—Epigallocatechin Gallate (EGCG) and Epicatechin Gallate (ECG)

**Figure 3:**
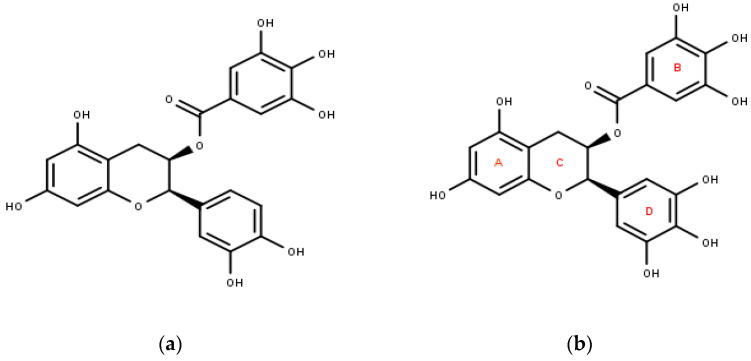
(**a**) Epicatechin gallate (IUPAC: [(2R,3R)-2-(3,4-dihydroxyphenyl)-5,7-dihydroxy-3,4-dihydro-2H-chromen-3-yl] 3,4,5-trihydroxybenzoate), (**b**) epigallocatechin gallate (IUPAC: [(2R,3R)-5,7-dihydroxy-2-(3,4,5-trihydroxyphenyl)-3,4-dihydro-2H-chromen-3-yl] 3,4,5-trihydroxybenzoate).

Flavanols are a sub-class of flavonoids; when present in monomeric form, they are often referred to as catechins, and their polymeric form is commonly called proanthocyanidins. The most studied and explored flavanols are catechin, epicatechin, epigallocatechin, gallocatechin, and their gallate derivatives. These are found in high concentrations in grapes (30–175 mg kg^−1^), green tea (100–800 mg kg^−1^), cocoa powder (c.1.4 g kg^−1^), chocolate (460–610 mg kg^−1^), and various berries (10–100 mg kg^−1^) [51,52]. The structure of EGCG involves three rings of aromatic nature—A, B, and D, which are linked to each other via a pyran ring (ring C). Its numerous health benefits are often associated with its structure. For example, its antioxidant activity is due to hydrogen transfer, which involves the B- and/or D-rings. (Figure 3) EGCG exhibits a variety of activities, including anti-inflammatory, anti-diabetes, anti-obesity, and anti-tumor activities [53].

EGCG is the constituent present in the highest quantity in green tea, and it is 70% of the catechin constituent. Epidemiological data have shown that green tea intake provides preventive and therapeutic actions against various chronic diseases, including cancer. Additionally, cell culture and animal studies have proven their anti-cancer potential and revealed different mechanisms of action, which exert the cancer-preventive effects of EGCG and green tea [54]. EGCG has shown anti-cancer activity via telomerase inhibition activity in numerous cancer cell line models, which are described below.

Numerous studies have proven EGCG’s anti-telomerase activity in breast cancer cells. EGCG and pEGCG, a novel pro-drug, have also inhibited the expression of hTERT, which is the catalytic subunit of telomerase responsible for telomerase activation in the human breast cancer cell model [55]. Similarly, EGCG and (−)-epigallocatechin (EGC) also downregulate the gene expression of hTERT, which promotes T47D breast cancer cells [56]. Studies have also reported the telomerase inhibition activity of MST-312, a chemical derivative of EGCG, on different cancer cells. A decrease in telomerase activity, the induction of telomere dysfunction, and growth arrest was observed in MDA-MB-231 and MCF-7 breast cancer cells [57]. Similar results were shown in MCF-7 cells by Zhang et al. and his associates [58]. MST-312 also induced apoptosis and inhibited telomerase activity in acute promyelocytic leukemia (APL) cells [59].

EGCG reduced telomerase activity in small lung cancer cells, and a decrease in caspase -3 and -9 was observed, thus showing initiation of apoptosis. Additionally, an analysis of SLCL cells indicated a cell cycle block in the S-phase [60]. EGCG has demonstrated anti-cancer activity in Ec9706 and Eca109 esophageal carcinoma cells, where altering the expression of caspase-3 protein and the telomerase activity level and membrane potential of mitochondria led to apoptosis [61]. In a study involving the nasopharyngeal cancer cell line CNE2, EGCG successfully decreased the mRNA and protein expression of hTERT and c-Myc protein, the overexpression of which causes cancer [62].

In cervical cancer studies, EGCG inhibited the activity of telomerase and the growth rate of endocervical (HEN-18) and ectocervical (HEC-18) cells [63]. Further, both OMC-4 and TMCC-1 cervical adenocarcinoma cell lines had their telomerase activity suppressed when exposed to EGCG, as determined by the telomeric repeat amplification protocol assay [64]. Cervical adenocarcinoma cells seemed to respond well to a combination of EGCG and retinoic acid, which caused apoptosis and stopped telomerase activity [65]. Another combination of EGCG and sulforaphane also induced apoptosis by downregulating hTERT and BCL-2 IN ovarian paclitaxel-resistant cancer cells [66]. EGCG and EGC have inhibited carcinoma cell growth by repressing hTERT transcription [67]. EGCG has significantly inhibited telomerase expression and has shown cytotoxic effects in 1321N1 and U87-MG glioma cells, where it also acts in conjugation with tamoxifen and cisplatin [68].

These studies (summarized in Table 2) demonstrate EGCG’s telomerase-inhibiting activities and suggest that it can function as a potent anti-cancer agent against diverse malignancies, as discussed above. Further detailed research is warranted to investigate its role in different types of cancer.

## 4. Flavones—Apigenin and Luteolin

### 4.1. Apigenin

**Figure 4:**
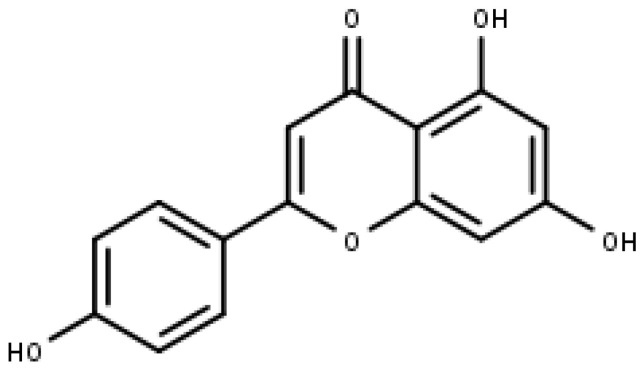
Apigenin (IUPAC: 5,7-dihydroxy-2-(4-hydroxyphenyl) chromen-4-one).

Apigenin (Figure 4) is a commonly found flavonoid belonging to the sub-class of flavones. It is found in significant amounts in fruits, vegetables, and beverages that are derived from plants, such as chamomile (3000 to 5000 μg/g), beer, red wine, onion, and parsley (45,035 μg/g), etc. It commonly occurs in apigenin-7-O-glucoside and different acylated derivatives in its natural source. Chemically, it is named 4′,5,7, -trihydroxyflavone, and structurally, it exists in the pure form of yellow needles [69,70].

Numerous biological properties attributed to apigenin have been reported, which include anti-inflammatory, anti-proliferative, antioxidant, and anti-carcinogenic properties. A screening investigation has reported that 160 different human cellular targets have been identified, demonstrating its anti-cancer activity. Furthermore, different mechanisms have also been established, supporting its anti-cancer therapeutic potential [71]. It also modulates some signaling pathways that play a role in cancer. Apigenin’s activity against cancer has been demonstrated in numerous in vitro cancer cells and in vivo animal models [72]. However, its mechanism of suppressing or inhibiting telomerase activity is not clearly known. Some studies have only been performed to show this activity.

Apigenin (100 µM) prevented cell growth, and telomerase activity was significantly diminished in leukemia cells, which further led to apoptosis [73]. The human telomerase reverse transcriptase (hTERT) is crucial in giving human malignant neuroblastomas immortality. Apoptosis up to 70% was induced in both SK-N-DZ and SK-N-BE2 cell lines; in addition, greater than 90% cell invasion inhibition was observed in malignant neuroblastoma cell lines (SK-N-DZ, SKN-BE2, SH-SY5Y, and IMR-32) [74]. The combination of apigenin (100 μM) and cisplatin was experimented with in triple-negative breast cancer (TNBC) MDA-MB-231 and HCC1806 cells. Concomitant to the combination’s telomerase inhibitory activity, downregulation of HTERT was also observed in both cells. Hsp90, p23, and other proteins that are important components of telomerase were also controlled by this combination [74]. Based on these findings, we can suggest that apigenin could be a potential telomerase inhibitor, warranting more studies and research.

### 4.2. Luteolin

**Figure 5:**
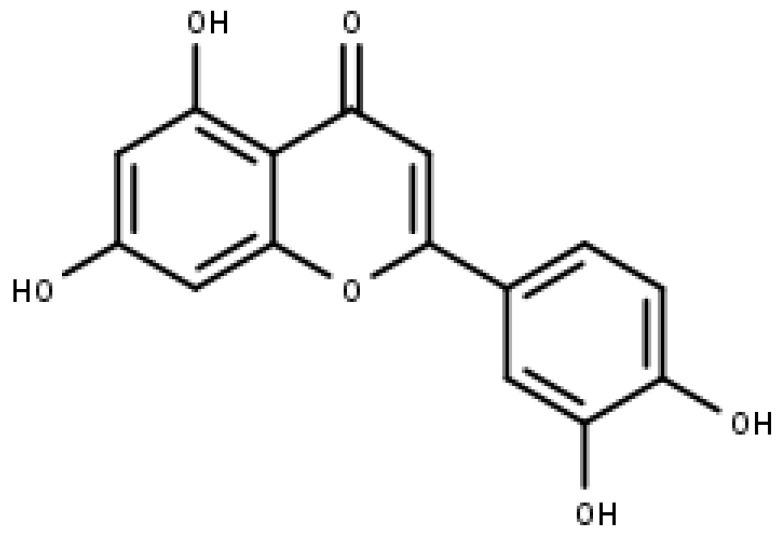
Luteolin (IUPAC: 2-(3,4-dihydroxyphenyl)-5,7-dihydroxychromen-4-one).

Luteolin (3,4,5,7-tetrahydroxy flavone) is another member of the sub-class of flavones, which is found in various dietary sources such as carrots, celery, olive oil, peppermint, rosemary, oregano, etc. It occurs naturally in its glycosylated form [75,76,77,78]. Two benzene rings (A and B) connected to an oxygen-containing pyrane ring (C) define the structure of the compound. (Refer Figure 5) The double bond between carbon atoms 2 and 3 and the four hydroxyl groups at positions 3′, 4′, 5, and 7 may all contribute to luteolin’s biological activity. Luteolin is believed to have several pharmacological actions, such as hepatoprotection, neuroprotection, and anti-cancer and anti-inflammatory properties, amongst others [79]. Its anti-cancer property is effective against different types of cancer, such as lung, glioblastoma, prostate, colon, pancreatic, and breast cancers [80].

Luteolin (10 and 30 µM) treatment causing S-phase cell cycle arrest was observed in MDA-MB-231 cells, followed by apoptosis. This effect was reported because of luteolin’s ability to suppress the expression of hTERT by reducing telomerase levels and the inhibition of the phosphorylation of NF-κB inhibitor α and c-Myc [81]. In-silico studies have also been reported, describing luteolin’s telomerase inhibition activity. Structural modifications were implemented in luteolin analogs and screened for their binding affinity to tankyrase II, which is an enzyme essential for telomere sustenance; 3 out of 15 analogs were reported to have comparable docking stores with luteolin [82]. Thus, this suggests that structural modifications could be key to developing active analogs, on which experimental studies should be conducted.

In conclusion, few experimental studies have demonstrated luteolin’s ability to inhibit telomerase and the related process; thus, its efficacy is unclear. To utilize luteolin to its utmost potential, detailed studies are required. With more trials, we will be able to further identify new cancer types where luteolin shows its telomerase-inhibiting activity.

## 5. Flavonols—Quercetin, Kaempferol, and Morin

### 5.1. Quercetin

**Figure 6:**
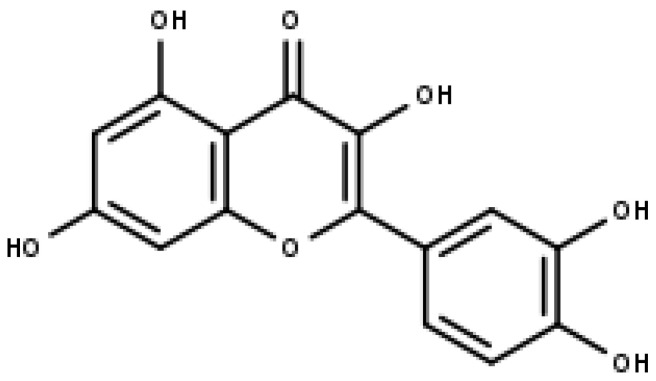
Quercetin (IUPAC: 2-(3,4-dihydroxyphenyl)-3,5,7-trihydroxychromen-4-one).

The flavonoid known as quercetin (3, 3′, 4′, 5, 7-pentahydroxyflavone) is a common substance that may be found in a wide variety of botanical and dietary sources. Propolis, along with other nutritious foods such as vegetables and fruits, particularly tea, apple (4.01 mg/100 g), broccoli (13.7 mg/100 g), and onion (45 mg/100 g), as well as red wine (3.16 mg/100 g), includes a significant amount of a flavonol called quercetin glycoside. Quercetin glycosides make up most of the total flavonoids in propolis. This substance has the presence of five hydroxyl groups at positions 3, 5, 7, 3′, and 4′ of the flavonoid (Figure 6) and is one of the characteristics that distinguish it as a unique substance [83,84,85]. A dietary additive is one possible use for quercetin. It has been shown that quercetin has a number of favorable benefits on human health, including anti-cancer properties, protection for the cardiovascular system, and anti-inflammatory activities. It is capable of acting as an anti-diabetic, anti-viral, anti-allergic, and anti-ulcer agent. Among its beneficial benefits are anti-infective, immuno-modulator, anti-hypertensive, and gastro-protective properties [86].

Investigations have shown that quercetin has qualities that are anti-cancer as well as those that inhibit the proliferation of cancer-causing cells and promote apoptosis. Quercetin is an established autophagy modulator that reduces cellular proliferation by causing cellular growth inhibition and cell migration and, ultimately, suppresses the growth of cancer cells by arresting the cell cycle. This is accomplished by inhibiting cell division during the colony formation stage [87,88]. Quercetin has been shown in a number of experiments to have substantial applications in the cancer treatment process as well as in the management of the disease. This is accomplished through quercetin’s ability to block telomerase activity and induce cell death [89,90,91].

Medications with estrogen receptor beta ligands, such as tamoxifen and quercetin, inhibit development and telomerase functioning in colorectal cancer. This is accomplished by preventing the tumor from dividing further [92]. According to the findings of a research paper that was published in 2001 by Choi et al., quercetin (20 µM) causes inhibition of growth in MCF-7 cell lines via two separate pathways. In the first place, quercetin (up to 20 µM) stops the cell cycle by causing a brief deposition in the M-phase, which is then accompanied by a stoppage in the G2-phase. Secondly, quercetin (20 µM) causes cell death [93].

Interestingly, the strategy that is connected with quercetin generating cytotoxic potency and apoptosis effects was identified in the human promyelocytic leukemia cell line (HL-60) and human lung cancer cell line by Kang and Seung-Eun [94]. They discovered that giving quercetin in greater quantities (up to 100 µM) entirely stopped cell growth when it was administered to the cells. In a study that was conducted in an identical fashion, Lee et al. (2006) detected the enhanced arrestation of the G2/M-phase of cell division, cell death, and DNA fragmentation in human leukemic monocyte lymphoma cells when quercetin was supplied to the cells. Regarding the investigation that was conducted on the effects of quercetin on apoptosis, Kou and Gibellini et al. discovered that quercetin (100–500 µM), in a dose-dependent manner, induces the arrest of cellular division at many phases, promotes cell death, and blocks the development of multiple cancerous cells [94,95]. Quercetin’s anti-carcinogenic and medicinal activities were also investigated in a number of in vivo experiments, which led to the discovery of the mechanisms underlying these benefits [96].

In addition, epidemiological research has found that consistent use of quercetin at doses ranging from 1.01 to 31.7 mg per day might lower the incidence of ovarian cancer [97]. In addition, investigations conducted both in vivo and in vitro revealed that quercetin had anti-cancer properties by preventing the revascularization and formation of tumors, arresting cells in the cell cycle, and triggering cell death [98,99]. Synergistic effects of quercetin and epigallocatechin gallate (EGCG) have been shown to have anti-tumor activity. These properties include the stimulation of the tumor suppression gene (p53), suppression of the cellular division pathway, caspase-induced apoptosis, and overexpression of death receptor-5 [100]. Avci et al. (2011) have demonstrated that quercetin (up to 100 µM) has apoptotic properties and anti-proliferative potency on cancer cells, including acute T-cell lymphoblastic leukemia, acute promyelocytic leukemia, and chronic myeloid leukemia [90]. It has been shown via this research that quercetin is an effective medicinal substance for the management of leukemia since it lowers the amount of telomerase activity as well as the amount of apoptosis-mediated cell death. In addition, quercetin inhibits the growth of numerous carcinoma cell lines. These cancer cell lines include those of the breast [101], laryngeal [102], nasopharyngeal [103], colon [104], and brain [105].

It has been shown via a number of studies involving cancer cell lines that quercetin has the capability of inhibiting the functioning of telomerase and inducing apoptosis (programmed cell death). Based on these findings, quercetin may have an impact that is anti-carcinogenic when it works via this particular route.

### 5.2. Kaempferol

**Figure 7:**
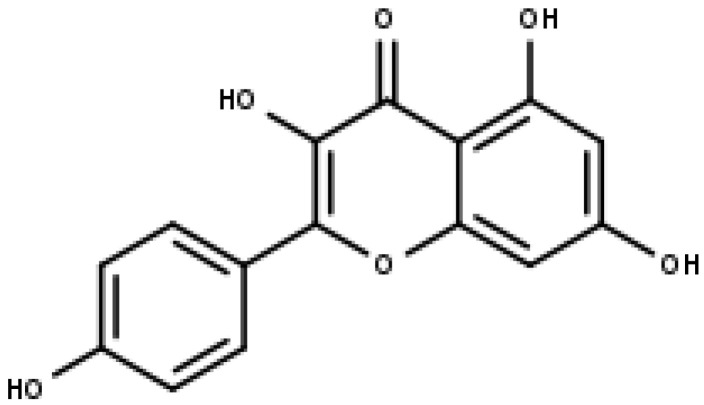
Kaempferol (IUPAC: 3,5,7-trihydroxy-2-(4-hydroxyphenyl) chromen-4-one).

Kaempferol is one of the aglycone flavonoids that are most frequently found in a glycoside state. It is a yellow chemical, and it is a kind of tetrahydroxyflavone where 4-OH moieties are situated at positions 3, 5, 7, and 4′ (Figure 7) [106]. The phytochemical kaempferol may be present in a variety of plant components, including vegetables, flowers, fruits, leaves, and seeds [107]. It has been shown that kaempferol and its glycoside analogs exhibit effects that are anti-tumor, anti-bacterial, antioxidant, anti-diabetic, anti-inflammatory, and neuroprotective. Kaempferol also has neuroprotective and cardioprotective properties [108].

According to the findings of epidemiological research, high consumption of kaempferol is connected with a lower risk of developing a variety of cancers, including cancers that may occur in organs such as the skin, liver, colon, ovary, pancreas, stomach, and bladder [109,110]. Within this framework, the use of kaempferol and associated applications in cancer treatment are garnering a significant amount of interest from the scientific community [109]. The majority of successful attempts in preventing cancer may be attributed to raising the rates of apoptosis, which works to suppress the development of cancer cells [111]. In fact, kaempferol is capable of inhibiting numerous cancer cells by inducing cell death, cell cycle inhibition at the G2/M-phase, the reduced expression of signal-transduction pathways, phosphoinositide 3-kinase (PI3K)/protein kinase B (AKT), and the expression of epithelial–mesenchymal transition (EMT)-related markers (N-cadherin, E-cadherin, Snail, and Slug) [112,113].

Kaempferol also stimulates the initiation of cysteine proteases involved in cell death commencement and implementation—caspases-3, -7, and -9 and poly (ADP-ribose) polymerase (PARP) [114]. As a result, kaempferol prevents the aggregation of reactive oxygen species (ROS) that are engaged in the progression of carcinoma. It has also been shown that kaempferol may prevent the formation of new blood vessels, in addition to its potential to maintain the vitality of regular cells [115].

### 5.3. Morin

**Figure 8:**
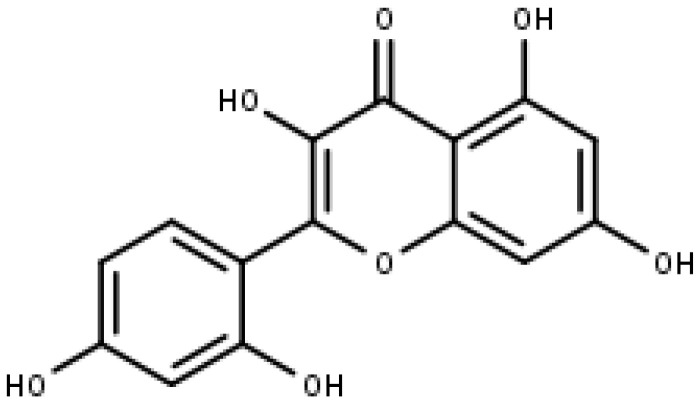
Morin (IUPAC: 2-(2,4-dihydroxyphenyl)-3,5,7-trihydroxychromen-4-one).

Morin is a polyphenol component that was first identified from a species of the Moraceae family, including mulberry figs and old fustic (Chlorophora tinctoria). The chemical formula for morin is 3,5,7,2′,4′-pentahydroxyflavone, which can be found in Figure 8. In previous research, it was demonstrated that morin (100 and 200 µM) inhibits the growth of a broad range of tumor cells in nude mice, particularly oral squamous cell carcinoma, leukemia, and COLO205 colorectal cancer cells [116].

Furthermore, the anti-cancer action of morin (50 µmol/L) is conducted via the suppression of transcription factors NF-B and STAT3 as well as the genes that are controlled by those transcriptional regulators. Through the stimulation of SHP1 protein tyrosine phosphatase, morin (50 µM) is able to block the phosphorylation mechanism of STAT3 at tyrosine-705 in tumor cells [117,118]. Its telomerase activity has also been seen when used in combination with MST-312. It is elaborated on in the subsequent sections.

## 6. Combinatorial Studies with Flavonoids and MST312

*MST-312 and quercetin:* Quercetin is a naturally occurring flavonoid that has been shown to have anti-proliferative effects against many different types of cancer. In addition to its impact on telomere shortening, the telomerase inhibitor MST-312 has been shown to have an anti-proliferative effect on many different types of cancer cells. Nevertheless, the therapeutic advancement of these substances is restricted since they have a poor absorption rate and are hazardous at greater dosages. In this work, we investigate the synergistic capability of their interaction in cancer cells, which might lead to a reduction in the therapeutic dose of these chemicals. This could be beneficial in the treatment of various cancers. According to our findings, MST-312 and quercetin display a potent synergism in ovarian cancer cells. When compared to treatment with either drug alone or with a vehicle, therapy with MST-312 and quercetin together increases the expression of DNA degradation and enhances apoptosis more so than treatment with either compound alone. In addition to this, we investigated the impact that these chemicals have on the rate of development of normal ovarian surface epithelial cells (OSEs). It is important to note that the combination of MST-312 and quercetin did not have any detectable effect on OSEs. This co-treatment selectively impacts cancer cells and decreases the effective dose of both medications; therefore, these discoveries have substantial implications for future efforts toward increasing the effectiveness of cancer therapies [119].

*Morin and MST-312:* One of the malignancies that are diagnosed most often across the globe is colorectal cancer, also known as CRC. In most cases, the malignant CRC that has already undergone metastasis in the advanced stage is resistant to the treatment that is now available and has a dismal outlook. However, effective targeted treatment for patients with metastatic colorectal cancer is not yet well established. We have conducted experiments to test the idea that a combination therapy consisting of the flavonoid morin and the telomerase inhibitor MST-312 might decrease the characteristics of cancer stem cells (CSCs). CD133/CD44 subpopulation profiling, the tumorsphere formation test, the cell invasion assay, and the wound healing assay were all carried out so that we could describe the CSC phenotype. In this study, the additive effects of the combination therapy of morin and MST-312 for 5-FU (5-fluorouracil) effectiveness in human colorectal cancer are investigated. In conclusion, the combination therapy of morin and MST-312 was successful in further enhancing the effectiveness of 5-FU and in chemo-sensitizing the 5-FU-resistant human colorectal cancer cells. When taken as a whole, the results of our research indicate that a new targeted treatment that combines the flavonoid morin with the telomerase inhibitor MST-312 may be able to enhance the prognosis of cancer patients [120].

## 7. Conclusions

Recent research has demonstrated telomerase as a potential target for anti-cancer therapy. Telomerase could work as a biomarker due to its presence in cancer cells and its absence in normal, unaffected cells. Thus, inhibiting telomerase and preventing cancer growth have been recently studied. Efforts have been made to develop synthetic telomerase inhibitors that can prevent telomerase expression in cancerous cells. However, in order to reduce side effects, many natural substances with numerous biological properties have often been tried for their telomerase suppression activity. As a major class of polyphenols, flavonoids are one group of compounds with anti-cancer properties. Due to this, their telomerase inactivation mechanism has been studied and experimented on. Thus, we conclude that common flavonoids, as described in this review, have successfully inhibited telomerase and its protein expression, thus demonstrating anti-tumor action in in vitro cell line studies and in in vivo studies to some extent. 

## 8. Future Perspectives

Certain challenges need to be addressed to make progress in this area. A fully known relationship between cancer and telomerase is yet undiscovered, thus preventing us from fully exploiting the use of flavonoids via this mechanism. Another limitation is that the effects of these substances are only on selected cancer types, thus suggesting that more research needs to be carried out in this field before using them for the therapeutic management of cancer. Similarly, more and more flavonoids should be tested and identified in order to learn more about the intricate relationship between the flavonoid mechanism and telomerase. Nevertheless, flavonoids’ possible role as telomerase inhibitors is an interesting field of investigation as anti-cancer therapy, and more research in this direction may lead to the development of novel treatment strategies that can specifically target telomerase activity in the malignant cells.

## Figures and Tables

**Table 1:** Recently completed clinical investigations targeting telomerase for cancer treatment.

Sr. No	Title	Study Type	Participants	Drug Used	Phase	Condition	Status	ClinicalTrials.gov Identifier	Reference
1	A Phase 1 Study of Imetelstat, a Telomerase Inhibitor, in Children with Refractory or Recurrent Solid Tumors and Lymphomas	Single Group Assignment	34 participants	Imetelstat sodium	I	Brain Tumor, Lymphoma, Lymphoproliferative Disorder, Small Intestine Cancer, Solid Tumor	Complete (October 2013)	NCT01273090	[40]
2	A Randomized Phase II Study of Imetelstat (GRN163L) In Combination with Paclitaxel (With Or Without Bevacizumab) in Patients With Locally Recurrent Or Metastatic Breast Cancer	Randomized, Parallel Assignment	166 participants	Imetelstat sodium (300 mg/m^2^), Bevacizumab (15 mg/kg), and Paclitaxel (90 mg/m^2^)	II	Locally Recurrent or Metastatic Breast Cancer	Complete (December 2012)	NCT01256762	[41]
3	A Phase II Trial to Evaluate the Activity of Imetelstat (GRN163L) in Patients with Essential Thrombocythemia or Polycythemia Vera Who Require Cytoreduction and Have Failed or Are Intolerant to Previous Therapy or Who Refuse Standard Therapy	Single Group Assignment	20 participants	Imetelstat (9.4 mg/kg)	II	Essential Thrombocythemia	Complete (April 2015)	NCT01243073	[42]
4	A Phase II Trial to Determine the Effect of Imetelstat (GRN163L) on Patients with Previously Treated Multiple Myeloma	Non-Randomized, Single Group Assignment	13 participants	Imetelstat (7.5 mg/kg, 9.4 mg/kg), Lenalidomide	II	Multiple Myeloma	Complete (November 2014)	NCT01242930	[43]
5	A Randomized Phase II Study of Imetelstat as Maintenance Therapy After Initial Induction Chemotherapy for Advance Non-small Cell Lung Cancer (NSCLC)	Randomized, Parallel Assignment	166 participants	Imetelstat (9.4 mg/kg) and Bevacizumab	II	NSCLC	Complete (September 2013)	NCT01137968	[44]

**Table 2:** Summary of the use of EGCG in different cell lines.

Flavonoid	Cell Line	Dose	Results	Reference
EGCG and pEGCG	MCF-7 and MDA-MB-231 breast cancer cell lines. MCF10A cell line (normal control)	Apoptosis induction and hTERT inhibition: EGCG (40 μmol/L) and pEGCG (20 μmol/L) Inhibition of cell proliferation: EGCG (60 μmol/L) pEGCG (40 μmol/L)	pEGCG demonstrated higher potency compared to EGCG in the inhibition of cell proliferation and apoptosis induction in breast cancer cell lines. Inhibition of hTERT was also shown in both cell lines.	[55]
EGCG	T47D breast cancer cells	80 µM	A significant decrease in hTERT gene expression causing apoptosis was observed.	[56]
MST-312 (derivative of EGCG)	MCF-7 and MDA-MB-231 breast cancer cell lines	0–10 µM	Reduction in telomerase activity, growth arrest, and induction of telomere dysfunction was observed in both cell lines, while reduced expression of TRF2 (telomere protective protein) in MDA-MB-231 cells.	[57]
MST-312	APL cells	0.5,1, and 2 µM	Caspase mediated apoptosis, arrest in G2/M-phase of the cell cycle of APL cells. Along with telomerase inhibitory activity, NF-κB activity was also suppressed. Additionally, hTERT, Bcl-2, survivin, Mcl-1, and c-myc genes were downregulated.	[59]
EGCG	SCLC cells (H69 and H69VP)	70 µM	50–60% Reduction in telomerase, 50 and 70% reduction in caspase 3 and 9, respectively, and block in the S-phase of the cell cycle was observed.	[60]
EGCG	Eca109 and Ec9706	100, 200, or 300 mg/L	EGCG produced apoptosis, reduced the mitochondrial membrane potential, and raised the expression of caspase-3 and led to the inhibition of telomerase.	[61]
EGCG	Nasopharyngeal carcinoma cell line CNE2	100, 200 µg/mL,	Prevented CNE2 cells from proliferating, caused cell cycle block, apoptosis of the cells was promoted, and downregulation of the mRNA and protein expression of hTERT as well as c-Myc protein.	[62]
EGCG	HEC-18, HEC-18T, HEN-18, HEN-18S	100 µM	Growth inhibition greater than 90% and induction of apoptosis was observed in HEC-18 and HEN-18. Telomerase was inhibited in all 4 cells.	[63]
EGCG	OMC-4 and TMCC-1	50 and 100 µM	Growth and telomerase inhibition, induction of apoptosis and pKi-67 suppression was observed in both cell lines.	[64]
EGCG and Retinoic Acid	HeLa and TMCC-1	EGCG: 100 µM RA: 1 µM	Combination treatment caused inhibition of telomerase, induction of apoptosis and prevented cell proliferation.	[65]
EGCG and Sulforaphane	SKOV3-ip1 and SKOV3TR-ip2 cells	20 mmol/L EGCG and 10 mmol/L SFN	Combination treatment led to ovarian cancer cell inhibition, arrest in cell cycle phase G2/M and S, induction of apoptosis and DNA damage, reduction in hTERT and DNA methyltransferase 1	[66]
EGCG and EGC	H1299, OECM-1, SAS, WRO, SK-Hep-1, and Hep-3B cells	10–40 µM	EGCG and EGC caused apoptosis and suppressed hTERT mRNA and promoter activity.	[67]
EGCG, Cisplatin, and Tamoxifen	1321N1 and U87-MG cells	EGCG (100 µM) Cisplatin (up to 50 µM) Tamoxifen (up to 20 µM)	Telomerase suppression activity was observed in both glioma cell lines when used in combination.	[68]

## Data Availability

Not applicable.
